# Colectomy and Acute Renal Failure: A Case Report with Unusual Presentation

**DOI:** 10.1155/2014/821970

**Published:** 2014-07-17

**Authors:** Osman Zikrullah Sahin, Cemil Bilir, Teslime Ayaz

**Affiliations:** ^1^Department of Internal Medicine, Recep Tayyip Erdogan University School of Medicine, Turkey; ^2^Portakallık Mh Melek Aprt. KAt 3 Daire 8 Rize Merkez, Turkey

## Abstract

Surgery is the only curative modality but occasionally it can have some long term complication such as short bowel syndrome. We presented a case reporting a 63-year-old man who had subtotal colectomy with liver metastasectomy according to the colon adenocarcinoma, following the couple of months of surgery; he had acute kidney injury without any end-organ damage while he had a regular diet and nutrition. Following the regular treatment of renal failure, colorectal cancer recurrence was excluded and then he was discharged from the hospital with a normal serum creatinine level. The patient was admitted to the nephrology clinic again for acute renal failure within 3 weeks of last admission to the hospital. He also denied the insufficient oral water intake and nutrition, but laboratory examination revealed acute renal failure. We suspected for short bowel syndrome (SBS). Following the hydration, loperamide hydrochloride 10 mg/day was started and the patient was followed up with normal serum creatinine and uric acid levels. To the best of our knowledge, this is the first case report, in which a patient with short bowel syndrome presented with prerenal acute renal failure even though he had sufficient oral intake and nutrition and can be treated with hydration and loperamide hydrochloride.

## 1. Introduction

Colorectal cancer (CRC) is a common as well as lethal disease. It is estimated that worldwide, in 2008, 1.23 million new cases of colorectal cancer were diagnosed [1]. Surgery is the only curative modality for localized colon cancer with a minimal morbidity [1]. Short bowel syndrome has many metabolic complications, but large intestinal resection has lower rate of metabolic abnormalities such as chronic salt and water depletion [2]. Excluding the surgical and oncologic outcomes, most of the colectomy patients can settle this situation. We present a case report with acute renal failure while the patient has an adequate nutrition and oral water intake.

## 2. Case 

**Figure 1:**
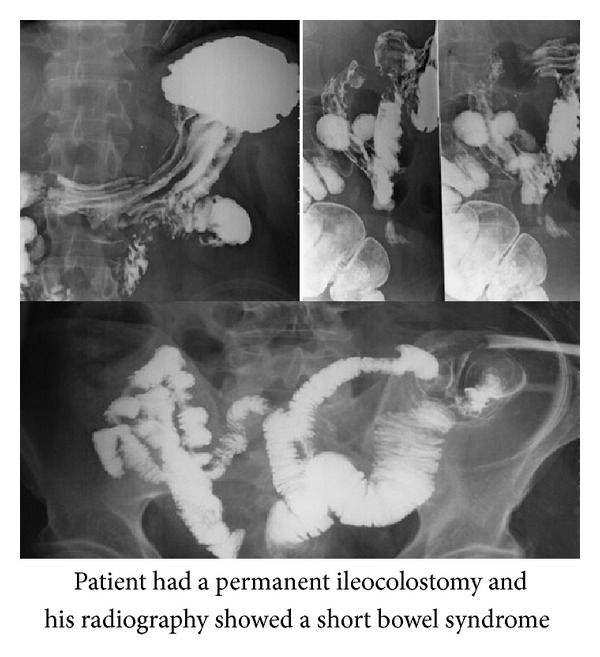
Radiological examinations demonstrated the short bowel syndrome.

A 63-year-old man had subtotal colectomy with liver metastasectomy in September 2012, according to the colon adenocarcinoma. In his surgery, 90% of colon was removed so he had a permanent ileocolostomy. The following couple of months after surgery, he had abdominal discomfort, diarrhea in his ostomy following the food intoxication, and acute kidney injury without any end-organ damage. While he had a regular diet and nutrition, in November 2013 he was admitted to the nephrology clinic with acute renal failure (serum creatinine 3.09 mg/dL, uric acid 13.5 mg/dL, and albumin 4.2 mg/dL with normal range of serum potassium, sodium, calcium, and phosphorus as well as serum hemoglobin and liver functions). Decreased skin turgor and dry oral mucosa were present on physical examination. Although he had the signs of dehydration, patient received 2 liters of water for each day and also he had at least 1 liter of urine output; he had no diarrhea and as stomal output. Intravenous hydration was started immediately and there was no acidosis on arterial blood gas analysis. 3000–4000 mL/day IV hydration improved the serum creatinine levels in 3 days (serum creatinine 1.3 mg/dL and uric acid 8 mg/dL), and changes of the laboratory parameters have been shown in Table 1. To exclude colorectal cancer recurrence, serum tumor markers such as CEA and CA 19-9 levels were measured and abdominal MRI and chest X-ray were performed. There were no findings of metastasis and recurrence on radiological and laboratory examinations. The patient was admitted to the nephrology clinic again for acute renal failure within 3 weeks of last admission to the hospital. He also denied the insufficient oral water intake and nutrition, but laboratory examination revealed acute renal failure (serum creatinine 5.0 mg/dL, uric acid 19.5 mg/dL, and albumin 4.0 mg/dL with normal range of serum potassium, sodium, calcium, and phosphor as well as serum hemoglobin and liver functions) and changes of the laboratory parameters have been shown in Table 1. Following the administration of intravenous fluid, serum creatinine and uric acid levels became in normal range within 4 days. After the second admission, we suspected for short bowel syndrome (SBS), and also radiological examinations demonstrated SBS (Figure 1). Loperamide hydrochloride 10 mg/day was started in the hospital and he had no side effects and serum creatinine levels did not increase in 5 days. We then discharged the patient and followed him up for the next 2 months. He had no symptoms for acute renal failure and his last serum creatinine level was 1.05 mg/dL and uric acid level was 7 mg/dL.

## 3. Discussion

To the best of our knowledge this is the first case report, in which a patient with short bowel syndrome presented with prerenal acute renal failure even though he had sufficient oral intake and nutrition. Short bowel syndrome (SBS) is a malabsorptive condition that can be caused by huge resection of the small intestine. SBS in adults usually results from surgical resection for Crohn's disease, malignancy, or radiation. The severity of clinical manifestations and symptoms is variable. In some cases of SBS, caloric and protein needs may be sufficient enterally, but vitamin and mineral deficiencies may still occur [3]. In many cases, the intestinal adaptation can permit transition to oral feeding. Intestinal failure is accepted as a new term, describing when gastrointestinal function is inadequate to maintain the nutrient and hydration status of the person without intravenous or enteral supplementation [4]. Chronic complications of SBS are liver and biliary disease related with parenteral nutrition, metabolic bone disease, arthritis, and colitis associated with bacterial overgrowth in intestine, enteric hyperoxaluria, and D-lactic acidosis [5]. Salt and water absorption is regulated in colon rather than small intestine; for this reason, we do not usually see acute renal failure in SBS [6]. In addition, decreases in NaCl absorption, resulting in impaired water absorption or water secretion, are the main electrolyte transport abnormalities in ulcerative colitis and Crohn's disease of the colon [7]. There is surprisingly little information available about the effects of segmental resection of the human colon on the ability of the remaining colon to absorb salt and water. Human proximal colon is the major site that has the greatest capacity for Na^+^, Cl^−^, and water absorption per unit area. In addition, descending colon and sigmoid colon normally make a relatively small contribution to the intact colon's overall capacity for salt and water absorption [6, 8]. Our case has a history of subtotal colectomy, and this may explain the prerenal failure even though he had sufficient oral hydration. According to these findings, we can speculate that adaptation to water reabsorption of small intestine and residual colonic segment may take 3–6 months following the surgery. In this period, we may prescribe loperamide hydrochloride if there is a renal failure risk.

In conclusion, this is the first case report in which a patient with short bowel syndrome presented with prerenal acute renal failure even though he had sufficient oral intake and nutrition. Oral loperamide hydrochloride may be used for SBS related prerenal acute renal failure.

## Figures and Tables

**Table 1:** Changes of the laboratory parameters during the admission.

Parameters	Before admission	Day 1	Day 3	After the discharge
First admission				
Serum creatinine, mg/dL	3.09	2.5	1.3	1.1
Blood urea nitrogen, mmol/L	24	20	7	6
Uric acid, mg/dL	13.5	11	8	6.5
Potassium, mEq/L	5.4	5	4.4	4.4
Second admission				
Serum creatinine, mg/dL	5	4.2	2.5	1.3
Blood urea nitrogen, mmol/L	32	25	15	7
Uric acid, mg/dL	19.5	14	11	7.5
Potassium, mEq/L	5.8	5	4.6	4.8
